# Supplementary material for: A strategy for differential abundance analysis of sparse microbiome data with group-wise structured zeros
Source: Sci Rep. 2024 May 30;14:12433. doi: 10.1038/s41598-024-62437-w (PMC11139916; doi:10.1038/s41598-024-62437-w)
Supplement: Supplementary file 1 — Supplementary Information. [file 41598_2024_62437_MOESM1_ESM.docx]

**Supplementary information to revisiting the impact of zero-inflation and structural zeros in differential abundance analysis of microbiome data**

**Text S1. Brief description on ZINBWaVe-based weighted and unweighted differential abundance methods**

**Statistical model**

The negative binomial (NB) model is used in popular software tools such as edgeR[^1^](https://www.zotero.org/google-docs/?4et0n9) and DESeq2[^11^](https://www.zotero.org/google-docs/?broken=ljXAat) for differential abundance analysis of count sequence data, taking into account over-dispersion. On the other hand, the zero-inflated negative binomial (ZINB) model simultaneously takes over-dispersion and zero-inflation into account. ZINB is a two-component mixture model with a degenerate distribution having a point mass at zero and a negative binomial (NB) distribution. The ZINB model for a count $y_{ij}$ from sample *i* and feature *j* with parameters: mean $\mu_{ij}>0$, feature specific dispersion $\theta_{j}>0$, and the probability of excess zero compared to NB $\pi_{ij}$ is given by

$$f_{ZINB}(y_{ij};\mu_{ij},\theta_{j},\pi_{ij})=\pi_{ij}\delta_{0}(y_{ij})+(1-\pi_{ij})f_{NB} (y_{ij};\mu_{ij},\theta_{j})$$

where $\delta_{0}(y_{ij})$ is the Dirac function and $f_{NB} (y_{ij};\mu_{ij},\theta_{j})$ is the negative binomial distribution with mean $\mu_{ij}$ and dispersion parameter $\theta_{j}$ given by

$f_{NB} (y_{ij};\mu_{ij},\theta_{j})= \frac{\Gamma(y_{ij}+\theta_{j})}{\Gamma(y_{ij}+1)\Gamma(\theta_{j})}\left( \frac{\theta_{j}}{\theta_{j}+\mu_{ij}} \right)^{\theta_{ij}}\left( \frac{\mu_{ij}}{\mu_{ij}+\theta_{j}} \right)^{y_{ij}}y_{ij}=0,1,2,\ldots$

ZINB is used in the ZINB-WaVE[^22^](https://www.zotero.org/google-docs/?broken=lz4Ohf) approach. In ZINB-WaVE modeling, the parameters $(\mu,\theta,\pi)$are allowed to adjust for both known and unknown sample level covariates and known feature-level covariates with a generalized linear model setting. The ZINB-WaVE model fitting uses a penalized maximum likelihood estimation procedure[^22^](https://www.zotero.org/google-docs/?broken=aKSCqU).

**ZINB-WaVE Weights**

For the purpose of computing ZINB-WaVE based observation weights[^21^](https://www.zotero.org/google-docs/?broken=SIv274), the parameters $(\mu,\theta,\pi)$are estimated in the ZINB-WaVE model with no latent variables inferred, which is equivalent to fitting a ZINB model. Using these estimated parameters, observation-specific weight or probability that a count $y_{ij}$ from sample i and feature j arising from negative binomial model can be computed by

$$\hat{w}_{ij}=\frac{(1-\hat{\pi}_{ij}) f_{NB} (y_{ij};\hat{\mu}_{ij},\hat{\theta}_{j})}{f_{ZINB}(y_{ij};\hat{\mu}_{ij},\hat{\theta}_{j},\hat{\pi}_{ij})}$$

These weights play an important role in downweighting excess zeros when used as observation-specific weights in the generalized linear models which are the underlying models of popular R packages such as edgeR, DESeq2 and limma-voom. For example, the ZINBWaVE-DESeq2[^21^](https://www.zotero.org/google-docs/?broken=gWTmkK) approach extends DESeq2 with a weighted version of negative binomial regression. For a single feature j, the model coefficients $\beta_{j}$ or log-fold changes, are estimated by maximizing a penalized weighted negative binomial log-likelihood of the form:

$$\hat{\beta}_{j}={argmax}_{\beta}\left( \sum_{i=1}^{n} \hat{w}_{ij}\cdot log(f_{NB} (y_{ij};\mu_{ij}(\beta),\theta_{j}))+ P(\beta;\lambda) \right)$$

where

$\hat{w}_{ij}$ are observation level weights estimated based on ZINBWaVE model ;

$\mu_{ij}(\beta)=S_{i}e^{\sum_{r=1}^{R} x_{ir}\beta_{r}}$ is the mean function with $S_{i}$ the normalizing scale factor for sample *i* and covariates $x_{ir}, r=1, ..., R$; and

$P(\beta;\lambda)=\sum_{r=1}^{R} \lambda_{r}\frac{{-\beta}_{r}^{2}}{2}$ is a ridge penalty term with penalty parameters $\lambda_{r}=\frac{1}{\sigma_{r}^{2}},r=1, ..., R$, specified for each covariate and $\sigma_{r}$ is estimated based on the ratio of empirical to theoretical upper quantiles of normal distributions[^11^](https://www.zotero.org/google-docs/?broken=aV7bPC). The inclusion of a ridge penalty term stabilizes the estimated model coefficients and their standard errors. This approach is implemented in the R package DESeq2.

The primary benefit of utilizing the edgeR and DESeq2 packages lies in their ability to estimate parameters using the ZINB-WaVE weights with a NB based weighted GLM approach. This is achieved by incorporating the weights directly into the "weights" slot of an object of class *DGEList* and *DESeqDataSet* in edgeR and DESeq2, respectively.

**Differential abundance methods**

**ZINBWaVE-DESeq2**

The DESeq2[^2^](https://www.zotero.org/google-docs/?QsB5Qy) package is extended to support the ZINBWaVE-DESeq2 approach for zero inflation by fitting NB weighted GLM for each feature using ZINB-WaVE observation level weights. To deal with zero inflation, the DESeq2 package accommodates the poscounts normalization from phyloseq package, which is based on geometric means of positive counts. The likelihood ratio test, which is suggested when the expected counts are very low, or the Wald test can be used for differential abundance testing.

**ZINBWaVE-edgeR**

The edgeR[^1^](https://www.zotero.org/google-docs/?5Q8r17) package is extended to support the ZINBWaVE-edgeR approach for zero inflation by fitting NB weighted GLM for each feature using ZINB-WaVE observation level weights. A moderated F-test is used to test differential abundance, similar to edgeR, but the residual degrees of freedom are adjusted as the sum of observation weights minus the number of parameters estimated by the NB weighted GLM.

#### **ZINBWaVE-limma-voom**

ZINB-WaVE-limma-voom is extended from the limma-voom[^23^](https://www.zotero.org/google-docs/?broken=wY0Yhj) approach implemented in the limma package[^24^](https://www.zotero.org/google-docs/?broken=WWYlM7). The limma-voom approach, which is based on weighted linear regression, uses heteroscedastic weights estimated from the mean-variance relationship of log-transformed counts[^23^](https://www.zotero.org/google-docs/?broken=SFsP2l). The ZINB-WaVE-limma-voom extension to account for zero inflation also uses limma-voom weighted linear regression but with weights obtained by multiplying the heteroscedastic weights from limma-voom with observation-level weights from the ZINB-WaVE model. Moderated test statistics can be used for differential abundance testing.

### **MaAsLin2**

The MaAsLin2[^20^](https://www.zotero.org/google-docs/?broken=DUQi49) approach uses a linear model to log-transformed abundance of each feature, with a pseudo count of 1 added to the abundance table to handle zeros and total sum scaling normalization as the default. The Wald test is used for significance testing.

#### **metagenomeSeq**

The metagenomeSeq[^18^](https://www.zotero.org/google-docs/?broken=b1Y0dK) approach uses a zero-inflated Gaussian mixture model for log-transformed abundance, with a pseudo count of 1 added to the abundance table to handle zeros. A sample-specific offset calculated using Cumulative Sum Scaling (CSS) normalization is used. A moderated t-test is used to identify differentially abundant features.

**ANCOM-BC**

ANCOM-BC[^3^](https://www.zotero.org/google-docs/?broken=g9ch3M) uses a linear regression framework based on log-transformed taxa counts, with a pseudo count of 1 added to the abundance table to account for sampling zeros and a sample-specific offset term for bias correction. ANCOM-BC identifies taxa due to structural zeros and performs no further analysis on them; instead, it labels them as differentially abundant. Taxa that do not have structural zeros are tested for differential abundance using standard statistical tests.

**Figures S1-S8**

**
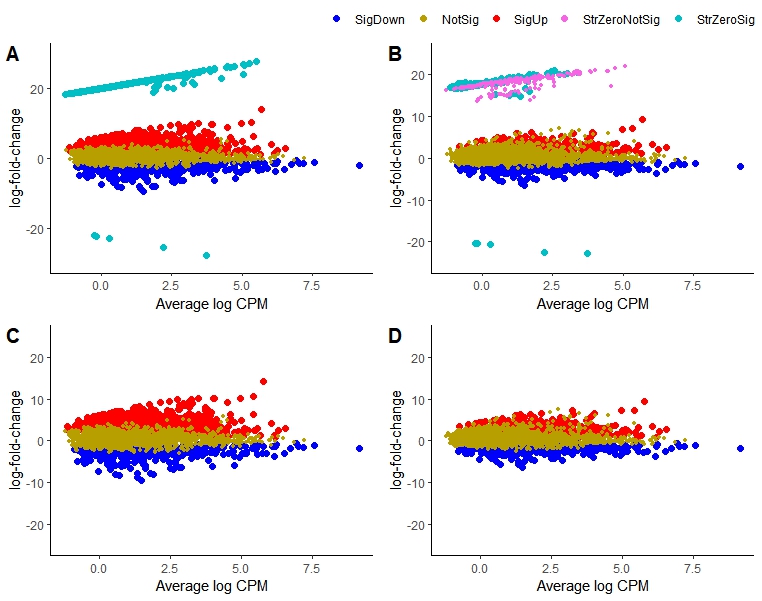
**

**Figure S1: Comparing differential abundance detection tools in the presence of perfect separation or structural zeros for Arctic fire soil.** SigDown: significant taxa with negative log-fold change, SigUp: significant with a positive log-fold change, NotSig: not significant, StrZeroSig: significant for taxa with structural zeros, StrZeroNotSig: not significant for taxa with structural zeros. A. Analysis with DESeq2, taxa with structural zeros found to be significant having relatively large log-fold changes and located on the boundary of the plot (cyan); B. Analysis with DESeq2-zinbwave, taxa with structural zeros found not to be significant (purple). The number of significant taxa identified by DESeq2 and DESeq2-zinbwave differed considerably due to the presence of taxa with structural zeros. C. Analysis with DESeq2 after excluding taxa with structural zeros; D. Analysis with DESeq2-zinbwave after excluding taxa with structural zeros.


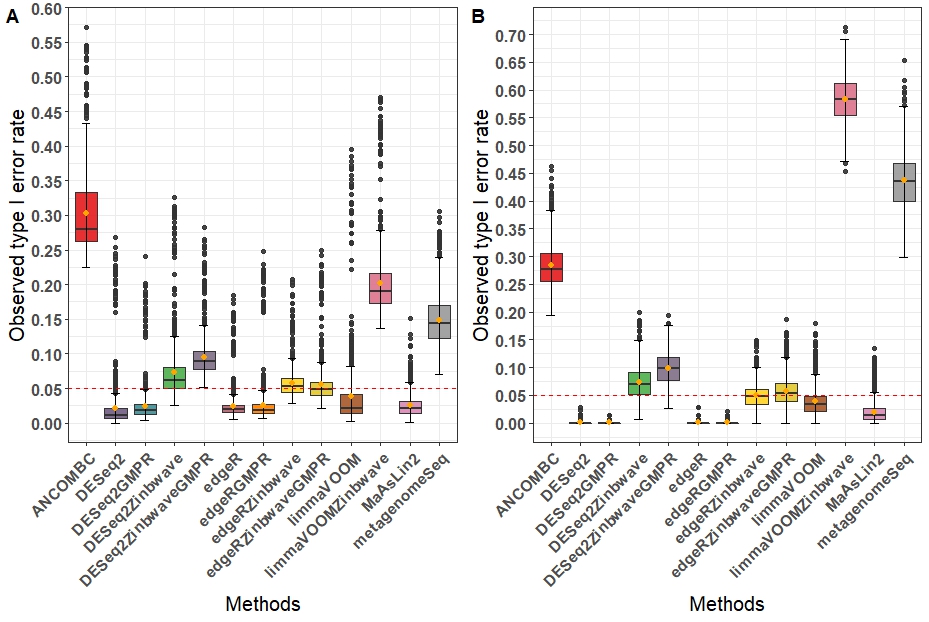


**Figure S2. Mock samples: controlling type I error rates using several differential abundance tools**. Unweighted and weighted differential abundance methods were evaluated for type I error control based on mock samples from plant microbiome data with varying zero-inflation rates. Left panel: N-P starvation template dataset with 55% zeros. Right panel: Forest-Potting soils template dataset with 75% zeros. Compared to the 5% nominal level, on average the observed type I error rates were very high for ANCOM-BC, metagenomeSeq, and limma-voom-Zinbwave; very low for DESeq2, edgeR and MaAsLin2; slightly higher for DESeq2-Zinbwave; and close to for edgeR-Zinbwave under the null hypothesis of no differentially abundant taxa.

**
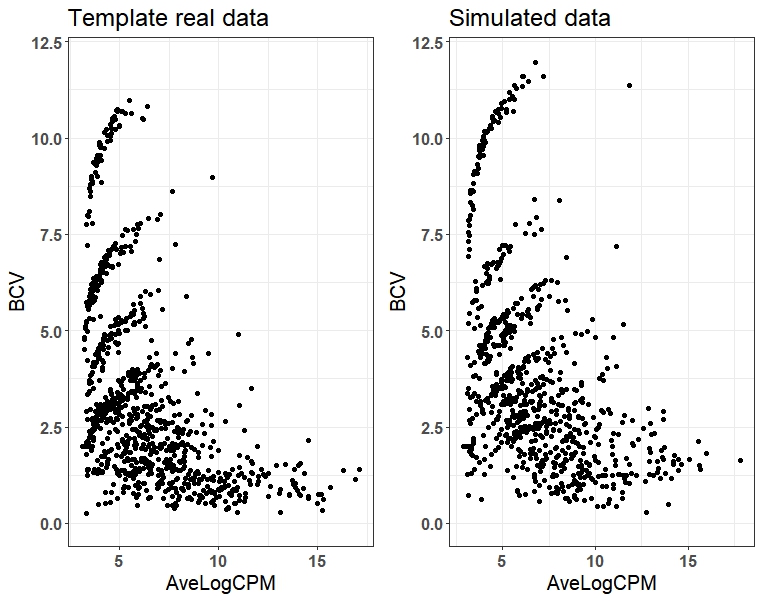
**

**Figure S3. Biological coefficient of variation (BCV) plots obtained from template real data and synthetic data**.The BCVs are displayed against the average number of log counts per million (AveLogCPM). The BCV plot displays striped patterns, which are a sign of taxa with many zeros and high estimates of dispersion. For the N-P starvation template real dataset, the BCV estimates from the template real data and one simulated data are comparable to each other.

**
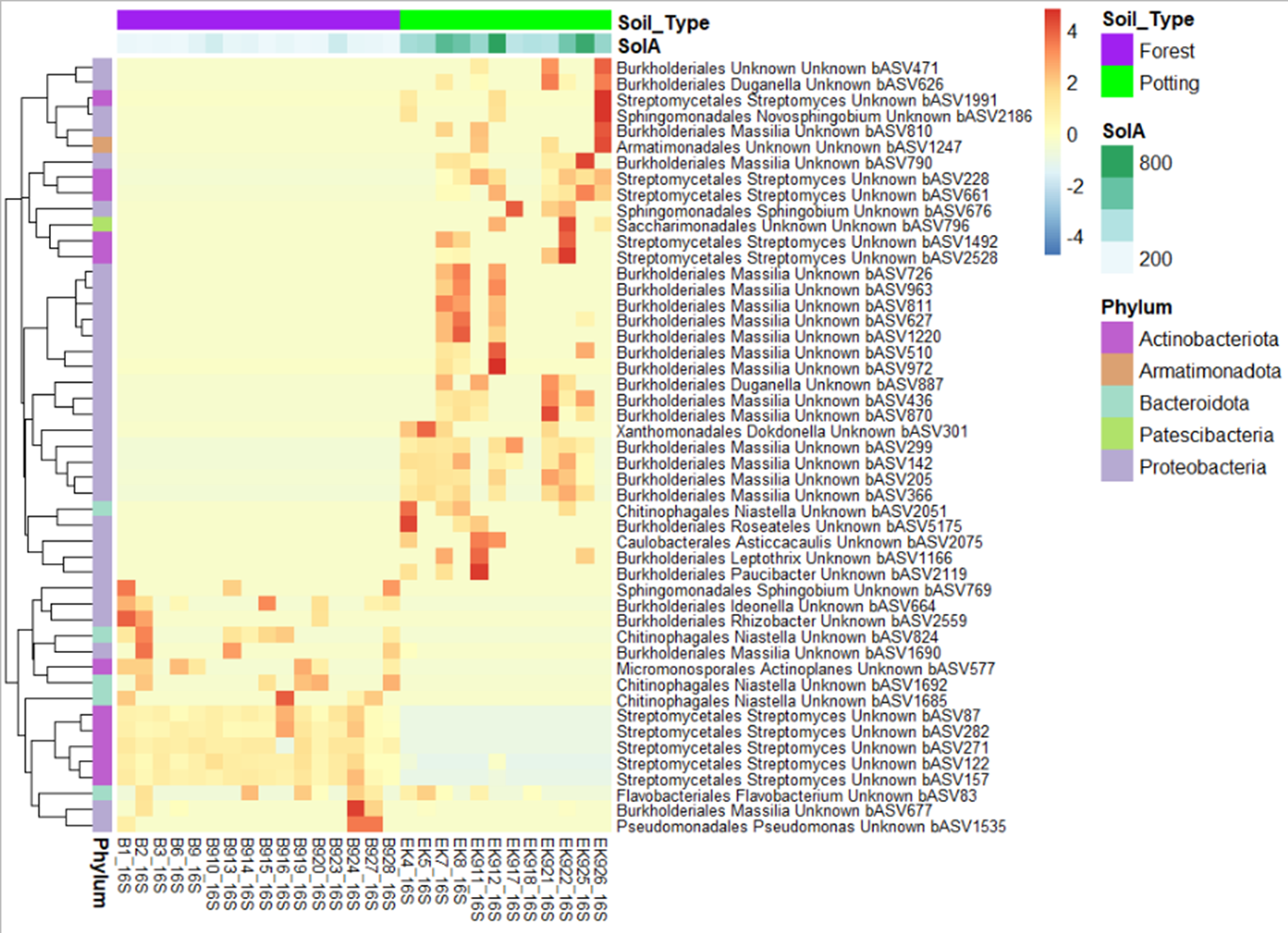
**

**Figure S4. Heatmap of differentially abundant taxa between potting and forest soils.** Significant taxa are shaded according to their relative abundance (dark red indicates high relative abundance) among the two soil types: Potting and Forest.


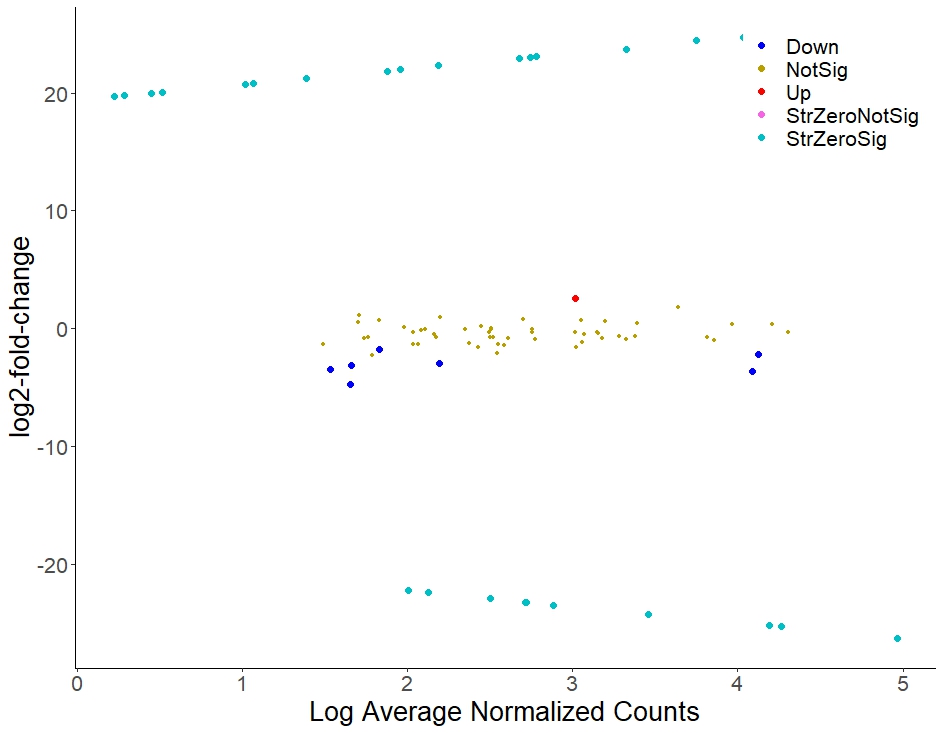


**Figure S5. Log2 fold change plot of all taxa analyzed for differential abundance between potting and forest soils.** Down: significant with a negative log-fold change (blue), Up: significant with a positive log-fold change (red), NotSig: not significant (golden), StrZeroSig: significant for taxa having structural zeros (cyan), StrZeroNotSig: not significant for taxa having structural zeros (purple). Taxa with structural zeros covers the top and bottom boundaries of the plot.


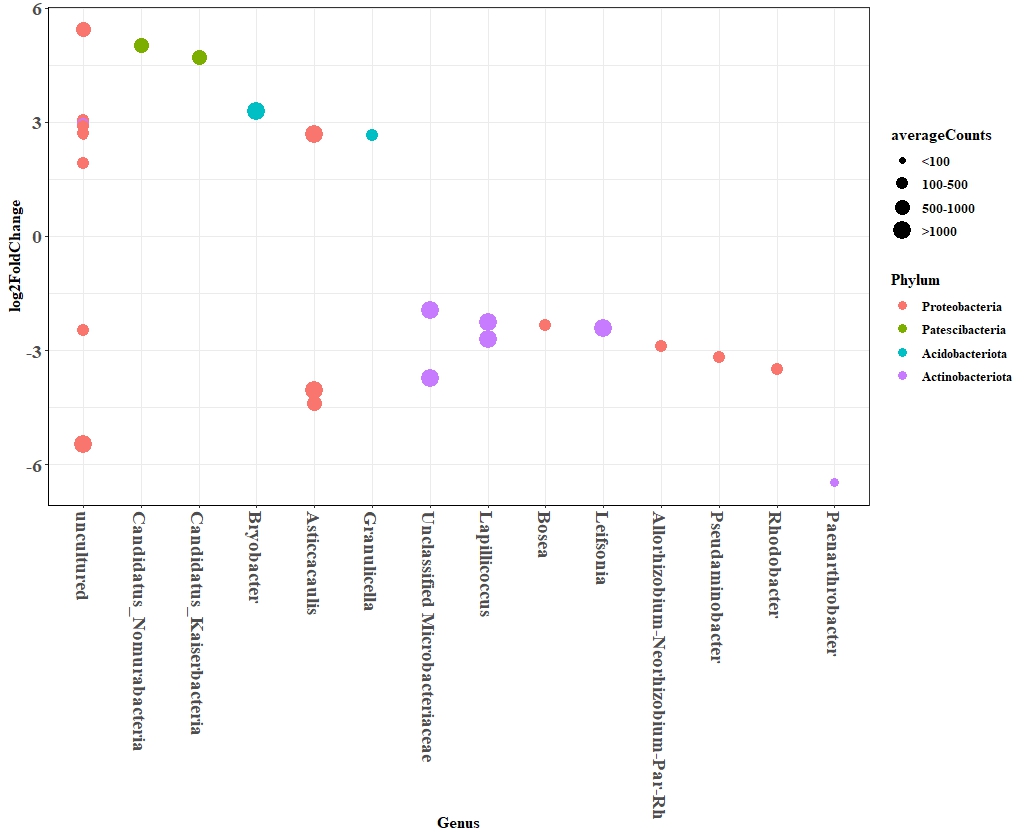


**Figure S6. Differentially abundant taxa having no structural zeros in comparing Phosphate deficiency with the control.**

**
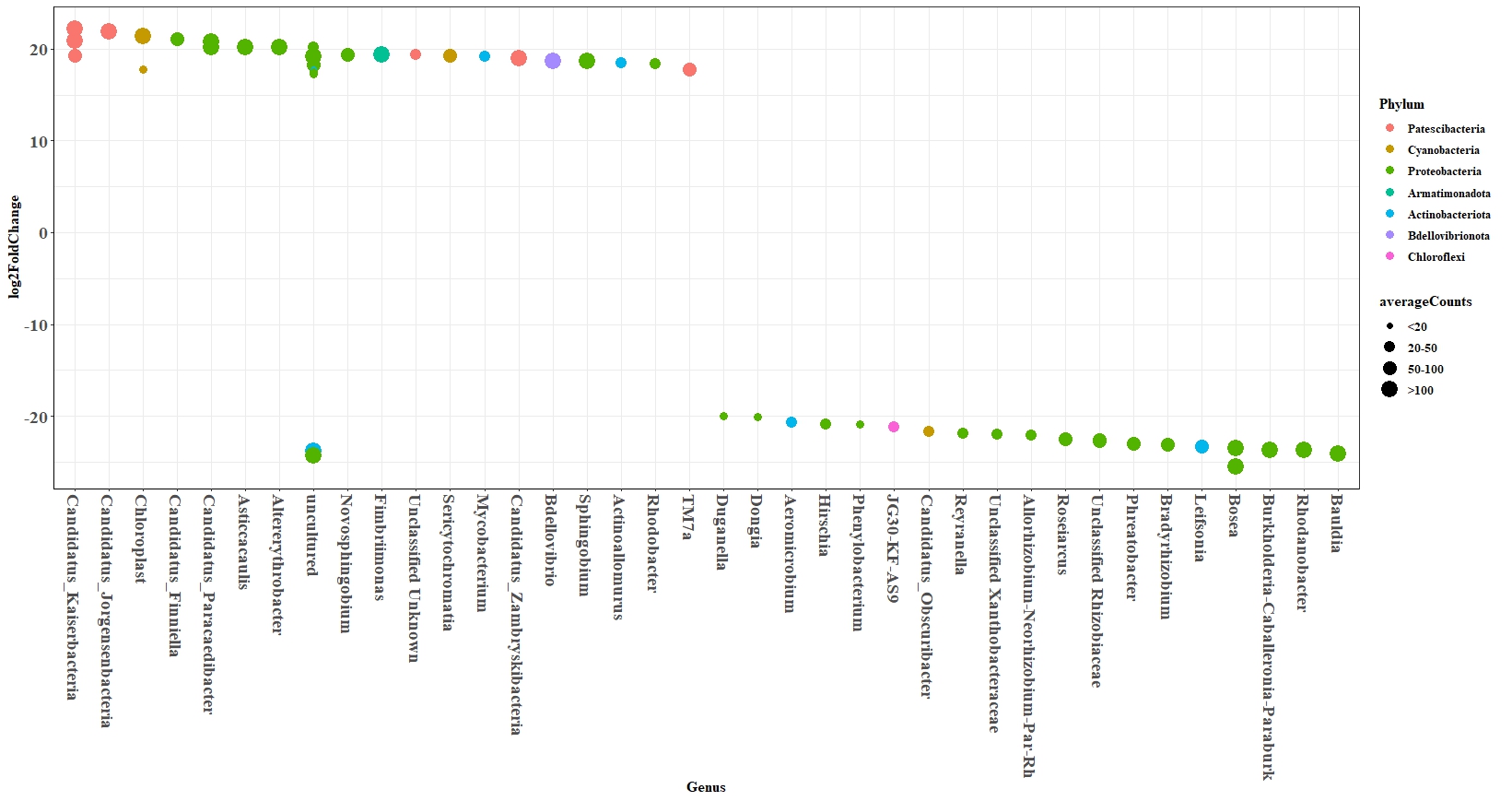
**

**Figure S7. Differentially abundant taxa having structural zeros in comparing Phosphate deficiency with the control.**

**Text S1. The potential of utilizing a combined DESeq2-Zinbwave and DESeq2 approach for differential abundance analysis**

The simulation findings showed that, after removing taxa with perfect separation or group-wise structured zeros, which allowed for a fair comparison of methods, DESeq2-Zinbwave is found to be most powerful as a differential abundance analysis technique for substantially as well as moderately inflated microbiome data. This can be reinforced further by analyzing taxa with group-wise structured zeros using the DESeq2 likelihood ratio test, a tool that performs penalized likelihood inference and is well-suited for assessing taxa with group-wise structured zeros. Therefore, as shown in Fig.1, we suggested to utilize a combined approach, which we refer to as DESeq2-DESeq2-Zinbwave that includes a differential abundance analysis of taxa with group-wise structured zeros using the DESeq2 likelihood ratio test and taxa without group-wise structured zeros using the DESeq2-Zinbwave likelihood ratio test.

To assess the performance of the combined approach DESeq2-DESeq2-Zinbwave, we reanalyzed the metagenome shotgun sequencing data from the Human Microbiome Project (HMP-2012), which include 5 supragingival and 5 subgingival plaque samples from the oral cavity [[9,35]](https://www.zotero.org/google-docs/?sFP9QA). The data were utilized in enrichment analysis to rank methods according to how well they could detect taxa that are known to be differentially abundant between the supragingival and subgingival plaques [[9]](https://www.zotero.org/google-docs/?Zvygwe). After filtering with a minimum of 10 non-zero counts in at least 2 samples, 516 taxa were retained, of which 94 had group-wise structured zeros and 322 did not. Based on genus-level metabolism, each taxon was classed as aerobic, anaerobic, facultative anaerobic, or unclassified [[9]](https://www.zotero.org/google-docs/?sASs8S). In comparing differential abundance of taxa between supragingival and subgingival plaques, it is expected to find an abundance of aerobic microbes in the supragingival plaque and of anaerobic microbes in the subgingival plaque. Fig. 6 shows the number of taxa found differentially abundant with adjusted p-value < 0.10 using DESeq2-DESeq2-Zinbwave (134), edgeR-edgeR-Zinbwave (89), DESeq2-Zinbwave (70), and edgeR-Zinbwave (38). In Fig. 6, we displayed the number of significant taxa belonging to aerobic and anaerobic metabolism only. In this figure, the number of taxa with positive log-fold changes that are more abundant in the supragingival plaque are indicated by the top bars whereas the number of taxa with negative log-fold changes that are more abundant in the subgingival plaque indicated by the bottom bars. The color of the bars represent aerobic (red) and anaerobic (blue) metabolism. The top red bars demonstrated that all four approaches correctly identified aerobic taxa in the supragingival plaque. Moreover, all of the approaches correctly (bottom blue bars) identified anaerobic taxa with varying numbers in the subgingival plaque but they also incorrectly (top blue bars) identified a few anaerobic taxa in the supragingival plaque. Further, similar to Calgaro and colleagues [9], we performed enrichment analysis based on Fisher's exact test (see details in [9]) to explore the relationship between supragingival and subgingival plaques and the growth of aerobic and anaerobic microbial species. Using Fisher's exact test (p-values for significant enrichment indicated on the bars), we found that all of the methods considered here correctly found an enrichment of aerobic microbes among the taxa over-abundant in supragingival plaque (red bars) and an enrichment of anaerobic microbes among the taxa over-abundant in subgingival plaque (bottom blue bars). However, Fisher's exact test revealed no significant enrichment of anaerobic microbes among taxa over-abundant in supragingival plaque (top blue bars in Fig. 6). In comparing the four methods, the methods can be ranked according to the number of accurately identified taxa. As a result, the combined approaches accurately discovered many more anaerobic microbes enriched in the subgingival plaque (long bottom blue bars). In particular, we found DESeq2-DESeq2-Zinbwave to be a more effective tool for discovering differentially abundant microbial species.


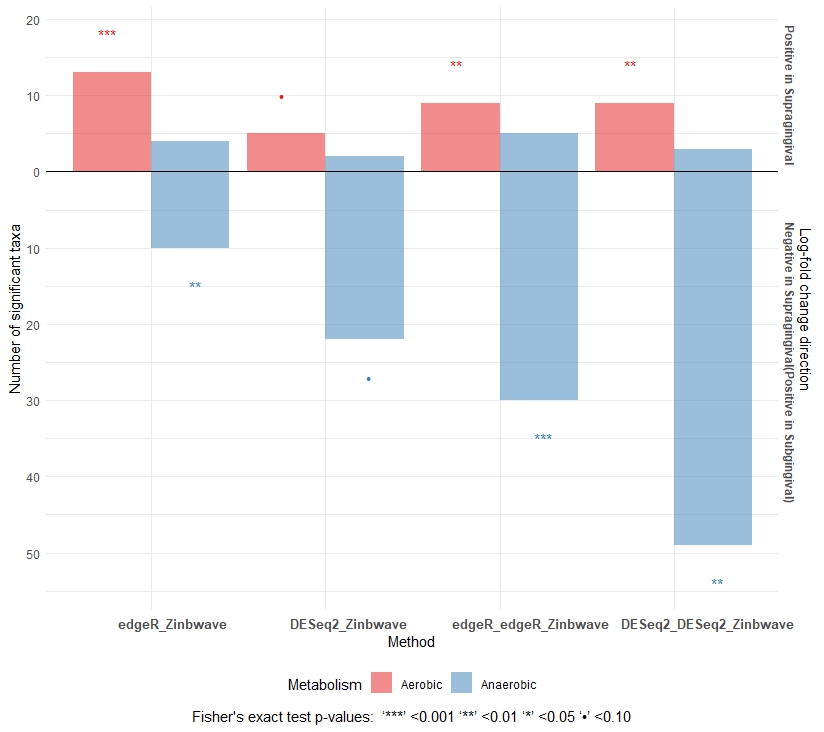


**Figure S8. Enrichment analysis comparing Supragingival vs Subgingival plaque for the metagenome shotgun sequencing samples from the Human Microbiome Project (HMP-2012).** Each bar represents the number of significantly (adjusted p-value < 0.10) abundant taxa by each method, with positive log fold changes in Supragingival (top red bars), negative log fold changes in Subgingival plaque (bottom blue bars), and positive log fold changes in Supragingival (top blue bars), coloured according to aerobic and anaerobic metabolism. A Fisher exact test is employed to establish the enrichment significance, and the p-values are shown.
